# Real-world evidence shows no consensus on PGT-A effectiveness depending on outcome denominator

**DOI:** 10.5935/1518-0557.20260056

**Published:** 2026

**Authors:** Jose G. Franco Jr, Claudia Petersen, Laura D. Vagnini, Fabiana C. Massaro, Bruna Petersen, Andreia Nicoletti, Juliana Ricci, Camila Zamara, Isabela M. Pasotti, Renata A. Pouza, Bianca C. Matuella, Elisange-la V. Espirito Santo, Joao B. Meziara, Antonio H Oliani, Joao Batista A. Oliveira

**Affiliations:** 1 Center for Human Reproduction Prof. Franco Jr, Ribeirão Preto, Brazil; 2 Paulista Center for Diagnosis - Research and Training, Ribeirão Preto, Brazil; 3 Department of Gynecology and Obstetrics, São José do Rio Preto School of Medicine (FAMERP), São José do Rio Preto, Brazil; 4 University of Beira Interior - Faculty of Health Sciences, Covilhã, Portugal; 5 Reproductive Medicine Unit - University Hospital Centre Cova da Beira, Covilhã, Portugal

**Keywords:** PGT-A, real-world evidence, live birth rate, outcome denominator, cycle initiated, post-treatment se-lection bias

## Abstract

**Objective:**

The routine use of preimplantation genet-ic testing for aneuploidy (PGT-A) remains controversial in assisted reproductive technology, particularly regarding its effectiveness in improving live birth rates. Evidence is derived from randomized controlled trials (RCTs), non-ran-domized studies, and real-world data (RWD). Although RCTs offer strong internal validity, they often lack exter-nal validity. Non-randomized studies may suggest associ-ations, but their lack of randomization limits causal infer-ence because of potential bias. RWD reflect routine clinical practice but are heterogeneous and prone to selection bias. A pooled evaluation restricted to RWD, supporting what is called real-world evidence (RWE), has not previ-ously been performed.

**Methods:**

A critical methodological issue is the de-nominator used to report live birth rate (LBR): per embryo transfer (ET), per oocyte retrieval (OR), or per cycle initiat-ed (CI). We performed a systematic review of RWD studies published between January 2020 and January 2026 using PubMed, Scopus, Google Scholar, and the Latin American Network Registration System. Studies were eligible if at least one arm included ≥800 cycles and reported live birth outcomes comparing PGT-A and non-PGT-A cycles.

**Results:**

Seven studies met the inclusion criteria, com-prising 70,816 live births in PGT-A cycles and 63,557 in controls. Reported LBRs ranged from 24% to 44% in non-PGT-A cycles and from 17% to 53% in PGT-A cycles. All studies reporting LBR per ET favored PGT-A. In contrast, studies reporting LBR per CI or OR favored the control group. The direction of effect was therefore fully explained by denominator choice.

**Conclusion:**

Real-world evidence does not demon-strate a consistent benefit of PGT-A, and the interpretation of effectiveness is strongly dependent on outcome denomi-nator selection. Therefore, the routine use of PGT-A should not be considered justified, and the medical literature has a duty to precisely define the subpopulations in which PGT-A might have value.

## INTRODUCTION

Preimplantation genetic testing for aneuploidy (PGT-A) has progressively become integrated into assisted reproductive technology (ART) with the objective of improving embryo selection and increasing live birth rates (LBRs). Recent position statements from major reproductive medicine societies have raised important concerns regarding the routine use of PGT-A. The Practice Committees of the American Society for Reproductive Medicine and the Society for Assisted Reproductive Technology (2024) noted that although the use of PGT-A has been increasing and the underlying technologies continue to evolve, its value as a routine screening test for all IVF patients has not been demonstrated. While earlier single-center studies suggested higher live birth rates in favorable-prognosis patients, more recent multicenter randomized controlled trials reported similar outcomes between PGT-A and conventional IVF when frozen embryo transfer is performed. The value of PGT-A in reducing clinical miscarriage also remains uncertain. Similarly, the European Society of Human Reproduction and Embryology concluded that currently available data show limited improvement in live birth rates with PGT-A and that claims of reduced miscarriage rates or shorter time to pregnancy in specific subgroups require further validation (ESHRE Add-ons working group, 2023). Finally, it concluded that PGT-A is currently not recommended for routine clinical use. Despite these cautious recommendations, routine PGT-A use continues to increase worldwide. While randomized controlled trials (RCTs) (Munné *et al*., 2019; Sato *et al*., 2019; Yan *et al*., 2021; Lin *et al*., 2025) offer strong internal validity, their strict eligibility criteria often limit external applicability. Real-world data (RWD) (SART, 2022; Zegers-Hochschild *et al*., 2023; 2025; 2026 [RedLARA 2020-2022]; Roberts *et al*., 2022; Ma *et al*., 2023; Sarkar *et al.,* 2023), in contrast, reflect routine clinical practice across heterogeneous populations.

An underexplored yet critical methodological issue in the PGT-A debate concerns the denominator used to calculate live birth outcomes. Live birth per embryo transfer (ET), per oocyte retrieval (OR), and per cycle initiated (CI) are not interchangeable metrics. They represent fundamentally different analytical frameworks. The present study evaluates whether discrepancies in the reported effectiveness of PGT-A in RWD studies are driven primarily by denominator selection rather than by biological superiority.

## MATERIALS AND METHODS

A systematic review of observational RWE studies published between January 2020 and January 2026 was performed. The databases searched included PubMed, Scopus, Google Scholar, and the Latin American Network Registration System. Eligible studies compared PGT-A and non-PGT-A cycles and reported live birth outcomes, with at least one study arm including ≥800 cycles.

Live birth rates were extracted according to denominator: ET, OR, or CI. Odds ratios (ORs) with 95% confidence intervals (CIs) were calculated or extracted when available. Importantly, denominator choice was treated as a structural analytical variable rather than as a secondary reporting detail.

## RESULTS

Seven large RWE studies were included, comprising 70,816 live births in PGT-A cycles and 63,557 in non-PGT-A cycles. A clear denominator-dependent pattern emerged: all ET-based analyses favored PGT-A, whereas CI/OR-based analyses favored controls.

Live birth rates ranged from 24% to 44% in non-PGT-A cycles (control) and from 17% to 53% in PGT-A cycles. Results were highly inconsistent across studies. All studies reporting live birth per ET favored PGT-A, whereas studies using CI or OR as denominators favored the control group. The choice of denominator fully explained the direction of effect. The results are shown in Table 1.

**Table 1:** Live birth outcomes in real-world studies comparing PGT-A and non-PGT-A cycles

Study	Outcome denominator	LBR Control (%) (n/N)	LBR PGT-A (%) (n/N)	Odds ratio (95% CI)	Direction of effect
SART (2022)	ET	41% (19,398/47,238)	53% (51,264/96,855)	0.61 (0.60-0.62)	Favors PGT-A
Zegers-Hochschild *et al*., 2023; 2025; 2026 (RedLARA 2020-2022)	ET	34% (9,245/26,870)	41% (6,589/16,101)	0.75 (0.72-0.78)	Favors PGT-A
Sarkar *et al*. (2023)	ET	43% (3,043/7,128)	49% (4,410/9,014)	0.78 (0.73-0.82)	Favors PGT-A
Ma *et al*. (2023)	CI/OR	31% (3,150/10,140)	22% (1,155/5,350)	1.64 (1.51-1.70)	Favors Control
Roberts *et al*. (2022)	CI/OR	44% (19,476/44,656)	27% (809/3,001)	2.10 (1.92-2.27)	Favors Control
Zegers-Hochschild *et al*., 2023; 2025; 2026 (RedLARA 2020-2022)	CI/OR	24% (9,245/38,572)	17% (6,589/38,381)	1.52 (1.47-1.57)	Favors Control

SART: Society for Assisted Reproductive Technology

RedLARA: Latin American Network of Assisted Reproduction

LBR: live birth rate

ET: embryo transfer

CI: cycle initiated

OR: oocyte retrieval.

## DISCUSSION

The present analysis demonstrates that the perceived effectiveness of PGT-A in real-world studies is fully aligned with denominator selection. When live birth is calculated per embryo transfer, PGT-A appears beneficial. However, when calculated per cycle initiated or per oocyte retrieval, the apparent benefit disappears or reverses. This phenomenon reflects what can be formally described as post-treatment selection bias. Reporting outcomes per ET conditions the analysis on a downstream event-successful embryo selection and transfer-thereby excluding cycles that fail to reach transfer because of the absence of euploid embryos. Statistically, this represents conditioning on a mediator influenced by the intervention itself.

In causal inference terms, ET-based reporting introduces collider stratification bias. By restricting the analysis to transferred embryos, the denominator becomes dependent on the intervention. Conversely, CI-based analysis reflects a patient-centered endpoint. From the patient’s perspective, the clinically relevant question is: “What is my probability of taking home a baby if I start this cycle?” The patient will need to know that they may undergo many more cycles, with the risk of failure (absence of blastocysts for analysis, all blastocysts being aneuploid, etc.), before obtaining a euploid embryo for transfer. This denominator-dependent inversion of effect suggests that much of the controversy surrounding PGT-A may stem from reporting conventions rather than from biological disagreement. The intervention may improve embryo-level efficiency while failing to improve cycle-level effectiveness. These findings raise important questions regarding the universal use of PGT-A. While selective use in specific populations (e.g., advanced maternal age or recurrent implantation failure) may be justified, routine application across all patient groups, particularly good-prognosis patients, requires stronger evidence demonstrating improved cumulative live birth per initiated cycle.

Economic implications must also be considered. PGT-A substantially increases treatment costs, including laboratory procedures, biopsy, genetic testing, and potential embryo vitrification. If cumulative live birth per cycle initiated is not improved, widespread routine application may increase the financial burden without proportional clinical gain. In publicly funded systems, this has direct implications for cost-effectiveness and the allocation of limited reproductive healthcare resources.

These findings have major implications for clinical counseling, policy decisions, and guideline development. Universal application of PGT-A in good-prognosis patients cannot be justified solely on the basis of ET-based metrics. Future studies should prioritize standardized reporting of cumulative live birth per initiated cycle, alongside transparent disclosure of transfer cancellation rates. Importantly, questioning universal application does not equate to rejecting the biological rationale of PGT-A. Rather, it calls for differentiation between embryo-level efficiency and cycle-level effectiveness. An intervention may optimize selection among embryos while failing to improve the ultimate patient-centered endpoint.

The consistency of denominator-driven directional shifts across independent data sets strengthens the methodological interpretation. Future research should prioritize cumulative live birth per cycle initiated, transparent reporting of transfer cancellation rates, and cost-effectiveness analyses stratified by prognosis group.

Limitations include the observational design and potential confounding. However, the consistency of denominator-driven directional shifts across independent data sets strengthens the methodological interpretation.

## CONCLUSION

Real-world evidence does not demonstrate a consistent improvement in live birth rates with PGT-A when analyzed using patient-centered denominators. The apparent benefit observed in ET-based analyses reflects structural statistical conditioning rather than unequivocal biological superiority. Standardization of outcome reporting is essential for the meaningful interpretation of PGT-A effectiveness. Studies analyzing the still unconfirmed value of PGT-A in increasing LBR in subpopulations (recurrent miscarriages, advanced age, etc.) should always precisely define the metric being analyzed and should prefer the use of LBR by CI or OR.
